# Subjective health status: an easily available, independent, robust and significant predictive factor at the prometaphase of vaccination programs for the vaccination behavior of Chinese adults

**DOI:** 10.1186/s12888-022-03830-5

**Published:** 2022-03-14

**Authors:** Zuxing Wang, Lili Chen, Jun Xiao, Fugui Jiang, Wenjiao Min, Shuyun Liu, Yunqiong Wang, Mengsha Qi

**Affiliations:** 1 Sichuan Provincial Center for Mental Health, Sichuan Academy of Medical Sciences & Sichuan Provincial People’s Hospital, Chengdu, 610072 China; 2 Key Laboratory of Psychosomatic Medicine, Chinese Academy of Medical Sciences, Chengdu, 610072 China; 3 The People’s Hospital of Wenjiang Chengdu, 86 Kangtai Road, Wenjiang District, Chengdu, 611135 China

**Keywords:** COVID-19, Vaccination behavior, Vaccination rate, Subjective health status, Related factors

## Abstract

**Background:**

The World Health Organization (WHO) proposed COVID-19 vaccination as an emergent and important method to end the COVID-19 pandemic. Since China started vaccination programs in December 2020, vaccination has spread to provinces and municipalities nationwide. Previous research has focused on people's vaccination willingness and its influencing factors but has not examined vaccination behavior. We examine the effectiveness of psychosocial factors in predicting vaccination behavior.

**Methods:**

A cross-sectional online survey was performed among Chinese adults on 8 May and 4 June 2021. The statistical analysis of the data included univariate analysis, receiver operator characteristics (ROC) analysis and ordinal multiclassification logistic regression model analysis.

**Results:**

Of the 1300 respondents, 761 (58.5%) were vaccinated. Univariate analysis showed that a high education level and good subjective health status were protective factors for vaccination behavior, while suffering from chronic diseases was a risk factor. ROC analysis showed that subjective health status (AUC = 0.625, 95% CI: 0.594–0.656, *P* < 0.001) was the best predictor of vaccination behavior. Logistic regression analysis with subjective health status as a dependent variable indicated that older age, female sex, depression, neurasthenia, obsession, hypochondriasis and chronic disease were significant risk factors, while positive coping tendencies were a significant protective factor.

**Conclusion:**

Our study found a simple and effective marker, subjective health status, that can predict vaccination behavior. This finding can guide future epidemic prevention work.

**Supplementary Information:**

The online version contains supplementary material available at 10.1186/s12888-022-03830-5.

## Introduction

The significant morbidity and death rates from the COVID-19 pandemic have also caused a global economic crisis [1]. The Centers for Disease Control and Prevention (CDC) and the World Health Organization (WHO) proposed COVID-19 vaccination as an emergent and important method to end the COVID-19 pandemic [2, 3]. Therefore, it has become a top priority for governments to vaccinate as many people as possible. Since China started vaccination programs in December 2020, vaccination has spread in various provinces and municipalities nationwide, and as of June 1, 2021, China reported 661,468,000 doses of COVID-19 vaccine [4]. However, this unprecedented mass vaccination also involves many difficulties in the era of the rapid dissemination of information on the internet.

To better guide vaccination programs, many studies in different periods and regions have focused on people's vaccination willingness and its related influencing factors [5–8]. For example, Wang et al. reported that among 806 nurses in Hong Kong, China, only 40.0% intended to accept COVID-19 vaccination from 26 February to 31 March 2020 [7]. Yoda et al. conducted internet research in September 2020 in Japan and found that 65.7% of 1100 participants showed willingness to be vaccinated [8]. Another study of 3646 respondents in Bangladesh from December 2020 to January 2021 showed that 74.6% of respondents indicated their intention to be vaccinated against COVID-19 when a safe and effective vaccine was available without a fee [5]. Recently, Zhao and Bishai examined the individual and state-level factors that contribute to both the intent to vaccinate and vaccination behavior in the U.S. [9]. There are too many variables in the transformation of willingness and intention into specific action; that is, even if an individual has the intention to be vaccinated against COVID-19, he or she may ultimately not be vaccinated for various reasons. Therefore, compared with vaccination willingness, the index of vaccination behavior is more intuitive, specific and practical in guiding vaccination programs. However, the participants studied in this research were all Americans [9]. As is well known, there are differences in epidemic prevention policies between China and the U.S., so the research conclusions obtained in either country may not be applicable in the other country, which is very important because it will profoundly affect the epidemic prevention policies of various countries. Moreover, there are very few studies focused on vaccination behavior, which is the ultimate goal of vaccination efforts.

At the same time, many psychosocial factors have been found to be significantly related to vaccination willingness, such as educational level [10], gender [5], age [5], residency [11], income level [12], the presence of chronic disease [5], marital status [13], subjective health status [14], mental health status [15] and coping styles [16]. Many studies have reported the vaccination willingness of patients with various chronic diseases. For example, 76.7% of 706 people in the United States living with multiple sclerosis were willing to be vaccinated [17]. Another study showed that 80.9% of Portuguese patients with multiple sclerosis were either definitely or probably willing to receive a COVID-19 vaccine [18]. A study in Bangladesh involving 506 patients with hypertension reported that 68% were willing to be vaccinated against COVID-19 [5], while the percentage of 244 diabetic patients was 61% [5]. Chan et al. reported that the intention to accept the COVID-19 vaccination among 660 cancer patients was only 17.9% [19]. From the above research, it can be seen that the vaccination intentions of patients with different chronic diseases, and even patients with the same disease, are very different. In fact, there are great differences in the severity of various chronic diseases, people’s views on chronic diseases and their psychological state. Subjective health status, another indicator reflecting the health status of respondents, can integrate the above variables and shows a stable and significant correlation with vaccination willingness [20]. Therefore, subjective health status, which focuses on the actual health perceptions of individuals, should be considered and studied in vaccination programs. It is also necessary to explore the social and psychological risk factors related to subjective health status.

Confronted with these gaps in knowledge, we aimed to examine the effectiveness of various psychosocial factors in predicting vaccination behavior. Based on the literature, we hypothesized that the subjective health status of individuals has the strongest predictive power in predicting their vaccination behavior. We also explore the risk factors associated with psychosocial elements that have the strongest ability to predict vaccination behavior.

## Materials and methods

### Study design and sample recruitment

A cross-sectional online survey was performed among Chinese adults aged 18–65 years from 8 May to 4 June 2021, the prometaphase of the vaccination program, to obtain the rate of vaccination behavior. We collected data through Wen Juan Xing (https://www.wjx.cn/vm/YIIyx1V.aspx), a professional online questionnaire survey platform that uses anonymous self-reports.

Filtering was used to exclude data from respondents who submitted incomplete or careless responses. Due to the length of our questionnaire, it took some time to complete it. Therefore, we excluded questionnaires completed in a short time (≤ 10 min). We assumed that participants who completed the questionnaire quickly were likely to fill in the questionnaire without carefully reading and understanding the items. In addition, the questionnaire could only be submitted after all the questions had been completed, so there were no missing values in our data set. However, we also excluded some examples of obvious logical errors in forward and reverse wordings. To encourage the subjects to complete the questionnaire, we gave each participant a financial reward. Finally, we excluded a total of 216 questionnaires.

Our sample was selected by Wen Juan Xing, which retained a number of potential subjects who agreed to participate in the investigation. Wen Juan Xing contacted and selected participants in China who met the sampling quota based on sex, age, geographical region and socioeconomic status. Therefore, in our study, adult participants aged 18–65 were representative of the above demographic factors. All the data sets received were automatically uploaded to the Wen Juan Xing platform at the end of the survey. This study was approved by the ethics committee of Wengjiang District People's Hospital of Chengdu (reference number: ec-2020–002). All participants provided written informed consent before completing the investigation. We confirmed that all methods were performed in accordance with the relevant guidelines and regulations.

### Measures

The respondents completed a battery of self-assessments that collected demographic information. For the Psychological Questionnaire on Emergent Public Health Events (PQEPHE) and Simple Coping Style Questionnaire (SCSQ) used in our study, we used McDonald’s ω to test the internal consistency [21]. Generally, values greater than 0.90 are excellent, those in the range of 0.80–0.90 are good, and values from 0.70–0.80 indicate acceptable reliability. Table 1 shows that according to the current data evaluation, most of the McDonald’s ω values generally had acceptable reliability.

**Table 1:** Characteristics of participants

Variable	Vaccinated (*n* = 761, 58.5%)	Not vaccinated (*n* = 539, 41.5%)	*P* value	McDonald’s ω	Total (*n* = 1300)
Sex (female)	370 (48.6%)	254 (47.1%)	0.595	-	624 (48.0%)
Age (years, mean, SD)	31.5 (8.9)	30.4 (7.8)	0.026*	-	30.1 (8.3)
Education	15.9 (1.4)	15.5 (1.7)	< 0.001*	-	15.6 (1.6)
Marital status			0.697	-	
Never married	295 (38.8%)	203 (37.7%)	-	-	498 (38.3%)
Divorced/Widowed	10 (1.3%)	10 (1.9%)	-	-	782 (60.2%)
Married	456 (59.9%)	326 (60.5%)	-	-	20 (1.5%)
Location of residence	761	539	0.633	-	
Urban	691 (90.8%)	485 (90.0%)	-	-	1176 (90.5%)
Rural	70 (9.2%)	54 (10.0%)	-	-	124 (9.5%)
Income (yuan, mean, SD)	21,204.8 (69,602.5)	17,876.9 (28,394.0)	0.294	-	19,824.0 (56,278.1)
Subjective health status			< 0.001*	-	
Very good	155 (20.4%)	74 (13.7%)	-	-	229 (17.6%)
Good	436 (57.3%)	286 (53.1%)	-	-	722 (55.5%)
Moderate	165 (21.7%)	164 (30.5%)	-	-	329 (25.3%)
Poor	4 (0.5%)	14 (2.6%)	-	-	18 (1.4%)
Very poor	1 (0.1%)	1 (0.2%)	-	-	2 (0.2%)
Chronic disease			< 0.001*	-	
Yes	45 (5.9%)	61 (11.3%)	-	-	106 (8.2%)
No	716 (94.1%)	478 (88.7%)	-	-	1194 (91.8%)
Positive coping tendency (SCSQ, mean, SD)	1.95 (0.4)	1.89 (0.4)	0.014*	0.73	1.92 (0.4)
Negative coping tendency (SCSQ, mean, SD)	1.26 (0.5)	1.3 (0.5)	0.014*	0.76	1.29 (0.5)
Depression (PQEEPH, mean, SD)	0.49 (0.5)	0.60 (0.6)	0.001*	0.78	0.54 (0.6)
Neurasthenia (PQEEPH, mean, SD)	0.55 (0.6)	0.67 (0.6)	< 0.001*	0.72	0.60 (0.6)
Fear (PQEEPH, mean, SD)	0.85 (0.6)	0.89 (0.6)	0.220	0.80	0.87 (0.6)
Obsession and anxiety (PQEEPH, mean, SD)	0.32 (0.5)	0.39 (0.5)	0.014*	0.71	0.35 (0.5)
Hypochondriasis (PQEEPH, mean, SD)	0.46 (0.6)	0.51 (0.6)	0.098	0.71	0.48 (0.6)

*Abbreviations*: *PQEEPH* Psychological questionnaire on an emergent event of public health, *SCSQ* simple coping style questionnaire. * is statistically significant

The PQEPHE was used to evaluate the psychological status of members of the public during the pandemic outbreak. The scale contains P1-P24, with 24 questions in total. It consisted of the following five dimensions: neurasthenia (P13, P16, P17, P18, P21), depression (P3, P5, P6, P7, P8, P11), hypochondriasis (P15, P20), fear (P1, P2, P9, P12, P14) and obsession (P4, P10, P19, P22, P23, P24). Items were scored on a four-point scale ranging from 0 = none to 3 = serious; a score of 0 indicated that the respondent did not have the relevant psychological problem, while a score of 1, 2 or 3 indicated that the level of the respondent’s psychological problem was mild, moderate or serious, respectively [22].

The SCSQ [23] consists of 2 dimensions, i.e., negative coping style and positive coping style, and has a total of 20 items that are scored from 0–3 each, where 0 is “not adopted” and 3 is “frequently adopted”. Items 1 to 12 measure a positive coping style, and the other items measure a negative coping style. The results show the average scores of the negative coping dimension and the positive coping dimension. Previous studies have shown that the scale has good reliability and validity [24, 25]. We also present the PQEPHE and SCSQ scales in Tables S1 and S2 of the supplementary materials.

### Statistical analysis

Descriptive statistical statistics are shown for the sample demographics. For continuous variables, the mean and standard deviation were reported, and for categorical variables, the percentage was reported. Each variable was divided into vaccinated and nonvaccinated groups for comparison. The chi-square test was used for categorical variables with expected counts greater than 5, and Fisher’s exact test was used for variables with expected counts less than 5. Independent-sample t-tests were used to compare the differences in continuous variables.

Because we only focus on the impact of a single factor on vaccination behavior, univariate analysis was performed with demographic variables and the outcome variable to examine the association between demographic variables and vaccination behavior. Univariate analysis was performed to calculate the crude odds ratio (COR) value through the logistic regression. When the influence of other confounding factors was not considered, that is, there was only one independent variable in the regression model, the value obtained was the COR. In the univariate analysis, we regarded nonvaccination as a risk factor. Therefore, when the COR value was greater than 1, it indicated that this factor would increase the risk of nonvaccination, and when the COR was less than 1, it indicated that this factor would reduce the risk of nonvaccination.

Then, receiver operator characteristic (ROC) analysis was conducted to evaluate the predictive ability of all factors with significant differences and associations between the vaccinated and nonvaccinated groups as estimated by the area under the curve (AUC). Finally, ordinal multiclassification logistic regression model analysis was performed to examine the association of sociodemographic variables and mental state with the strongest predictor of vaccination behavior obtained by ROC analysis. For all analyses, the significance level was set at p < 0.05. Statistical Product and Service Solutions software version 23.0 (SPSS Science, Chicago, IL, USA) was used for all the analyses.

## Results

Of the 1300 respondents, 761 (58.5%) were vaccinated, 624 (48%) were female participants, and the average age was 30.1 years. The average education level of vaccinated respondents was 15.9 years, and that of nonvaccinated respondents was 15.5 years; there was a significant difference between the two groups (*p* < 0.001). The subjective health status of vaccinated participants was significantly better than that of nonvaccinated participants (*p* < 0.001). The same trend was also found for chronic diseases (*p* < 0.001), age (*p* = 0.026), positive coping tendency (*p* = 0.014), negative coping tendency (*p* = 0.014), depression (*p* = 0.001), neurasthenia (*p* < 0.001) and obesity and anxiety (*p* = 0.014). The details of the above outcomes, marital status, income and location of residence are shown in Table 1.

Next, univariate analysis indicated that a high education level was associated with higher vaccination behavior (COR = 0.88, 95% CI: 0.82–0.96, *P* = 0.002). Respondents who reported the presence of chronic disease were significantly (reference: no chronic disease, COR = 0.66, 95% CI: 0.45–0.86, *P* = 0.041) more likely to decide not to be vaccinated. At the same time, the level of subjective health status of respondents was good, which was a positive factor for vaccination behavior compared with a very poor level (COR = 0.62, 95% CI: 0.04–1.26, *P* = 0.015). The details of the univariate analysis are shown in Table 2.

**Table 2:** Univariate analysis

Variable	Crude odds ratio	95% CI	*P* value
Sex (reference: female)	1.02	0.81–1.29	0.882
Age	1.01	0.99–1.03	0.398
Education	0.88	0.82–0.96	0.002*
Marital status (reference: divorced/widowed)			
Never married	0.93	0.35–2.45	0.885
Married	0.92	0.36–2.34	0.862
Location of residence (reference: rural)			
Urban	1.05	0.71–1.57	0.798
Income	1.00	1.00–1.00	0.684
Subjective health status (reference: very poor)			
Very good	0.48	0.03–1.05	0.307
Good	0.62	0.04–1.26	0.015*
Moderate	0.83	0.05–1.87	0.296
Poor	1.97	0.10–1.83	0.360
Chronic disease (reference: yes)			
No	0.66	0.45–0.86	0.041*
Positive coping tendency	0.79	0.59–1.06	0.117
Negative coping tendency	1.21	0.95–1.55	0.131
Depression	1.19	0.85–1.67	0.306
Neurasthenia	1.22	0.89–1.68	0.211
Fear	0.93	0.71–1.22	0.581
Obsession and anxiety	0.86	0.55–1.35	0.510
Hypochondriasis	1.07	0.83–1.38	0.621

*Abbreviations*: *CI* Confidence interval, *PQEEPH* Psychological questionnaire on an emergent event of public health, *SCSQ* Simple coping style questionnaire

**Fig. 1:**
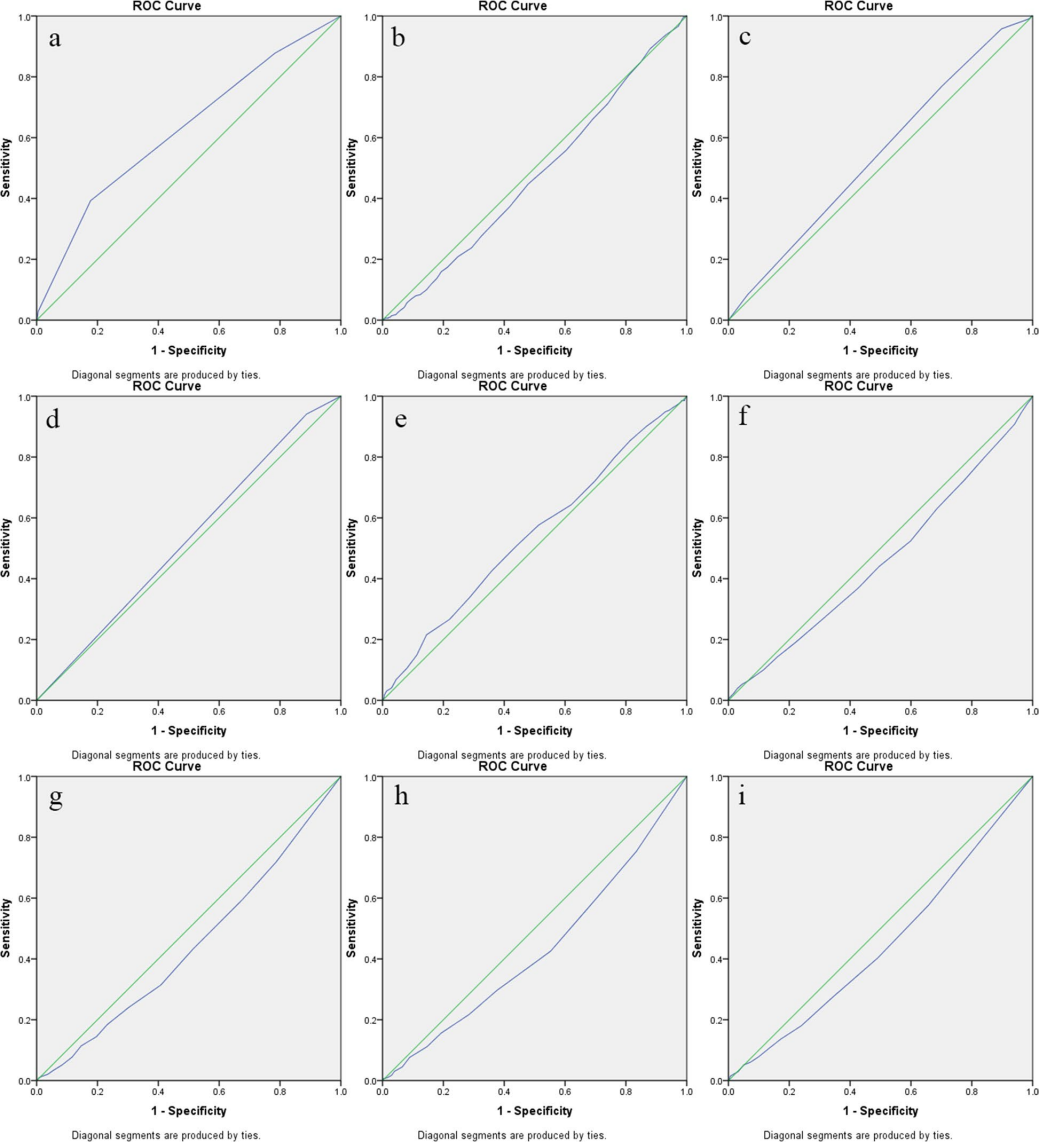
ROC curve of significant elements in predicting the vaccination behavior. **a** ROC curve of subjective health status for predicting vaccination behavior, **b** ROC curve of age for predicting vaccination behavior, **c** ROC curve of education for predicting vaccination behavior, **d** ROC curve of chronic diseases for predicting vaccination behavior, **e** ROC curve of positive coping tendency for predicting vaccination behavior, **f** ROC curve of negative coping tendency for predicting vaccination behavior, **g** ROC curve of depression for predicting vaccination behavior, **h** ROC curve of neurasthenia for predicting vaccination behavior, **i** ROC curve of obsession and anxiety for predicting vaccination behavior

ROC analysis showed that subjective health status was the strongest predictor of vaccination behavior among all factors (AUC = 0.625, 95% CI: 0.594–0.656, *P* < 0.001). The predictive ability of other factors with significant differences between the vaccinated and nonvaccinated groups was as follows: age (AUC = 0.472, 95% CI: 0.440–0.504, *P* = 0.083), education (AUC = 0.544, 95% CI: 0.513–0.576, *P* = 0.006), chronic diseases (AUC = 0.527, 95% CI: 0.495–0.559, *P* = 0.097), positive coping tendency (AUC = 0.541, 95% CI: 0.509–0.572, *P* = 0.013), negative coping tendency (AUC = 0.460, 95% CI: 0.428–0.492, *P* = 0.014), depression (AUC = 0.442, 95% CI: 0.411–0.474, *P* < 0.001), neurasthenia (AUC = 0.430, 95% CI: 0.399–0.461, *P* < 0.001) and obsession and anxiety (AUC = 0.448, 95% CI: 0.416–0.480, *P* = 0.001). Figure 1 shows the ROC curve for the ability of all significant elements to predict vaccination behavior.

Furthermore, ordinal multiclassification logistic regression model analysis showed that age (OR: 1.05, 95% CI: 1.03–1.06), sex (male vs. female; OR: 1.35, 95% CI: 1.09–1.68), depression (OR: 1.56, 95% CI: 1.14–2.15), neurasthenia (OR: 1.53, 95% CI: 1.13–2.15), obsession and anxiety (OR: 1.59, 95% CI: 1.38–1.91) and hypochondriasis (OR: 1.75, 95% CI: 1.59–1.96) were significant risk factors for worse subjective health status. However, positive coping tendency (OR: 0.57, 95% CI: 0.43–0.75) and chronic condition (yes vs. no; OR: 0.27, 95% CI: 0.18–0.40) were significant elements associated with better subjective health status (Table 3).

**Table 3:** Results of ordinal multiclassification logistic regression model analysis concerning subjective health status as a dependent variable

	OR	95% CI	*p*
Gender (reference: male)			
Female	1.35	1.09–1.68	0.007*
Marital status (reference: divorced/widowed)			
Never married	1.21	0.49–3.00	0.686
Married	1.61	0.67–3.86	0.295
Chronic disease (reference: yes)			
No	0.27	0.18–0.40	< 0.001*
Location of residence (reference: rural)			
Urban	0.99	0.68–1.45	0.983
Age	1.05	1.03–1.06	< 0.001*
Education	0.97	0.88–1.08	0.615
Income	1.00	1.00–1.00	0.208
Positive coping tendency (SCSQ)	0.57	0.43–0.75	< 0.001*
Negative coping tendency (SCSQ)	0.98	0.77–1.23	0.832
Depression (PQEEPH)	1.56	1.14–2.15	0.006*
Neurasthenia (PQEEPH)	1.53	1.13–2.15	0.006*
Fear (PQEEPH)	1.09	0.84–1.41	0.508
Obsession and anxiety (PQEEPH)	1.59	1.38–1.91	0.016*
Hypochondriasis (PQEEPH)	1.75	1.59–1.96	0.022*

*Abbreviations*: *PQEEPH* Psychological questionnaire on an emergent event of public health, *SCSQ* simple coping style questionnaire. * are statistically significant

## Discussion

To the best of our knowledge, this is the first study on COVID-19 to take vaccination behavior as a primary outcome index and analyze its predictors. The survey was performed during May and June 2021, at a time when vaccination projects were actively promoted across China. From our online survey, we found that 58.5% of respondents had been vaccinated, and there were significant differences in subjective health status, age, education level, chronic diseases, positive coping tendency score, negative coping tendency score, depression, neurasthenia and obesity between vaccinated and nonvaccinated people. In addition, a high education level and good subjective health status were protective factors for vaccination behavior, while suffering from chronic diseases was a risk factor. ROC analysis showed that subjective health status was the best predictor of vaccination behavior among all factors. Ordinal multiclassification logistic regression analysis with subjective health status as a dependent variable indicated that older age, female sex, depression, neurasthenia, obsession, hypochondriasis and chronic disease were significant risk factors and that positive coping tendency was a significant protective factor.

Previous studies on COVID-19 have generally shown that factors such as worse self-reported health, chronic diseases and older age were protective factors for the vaccination rate [26]. The same trend was observed in the initial outbreak of COVID-19 and the initial stage of vaccination [8, 14, 27], but the trend gradually changed with the vaccination program in various countries. With the development of COVID-19 vaccination work, the vaccination intention of people with chronic diseases has become not significantly different from that of healthy people [28]. Even in elderly people, vaccination intention has become significantly lower than among younger people [5]. However, it should be noted that our research indicated that in terms of the current vaccination rate, vaccinated people are significantly older than unvaccinated people, which may indicate the cumulative effect of the high vaccination behavior of elderly people. Even if the vaccination intention of elderly people is gradually decreasing, their vaccination rate is still higher than that of the younger age group.

Our study shows that the prevalence of chronic diseases in the nonvaccinated population is significantly higher than that in the vaccinated population, and univariate analysis indicates that suffering from chronic disease is a risk factor for vaccination behavior. Vaccination against COVID-19 has been considered to be one of the most promising and cost-effective health interventions for the prevention and control of the pandemic. The vaccination situation of the COVID-19 vaccine has been widely reported by the mass media and has attracted a high degree of attention in the population. Therefore, any possible negative news about vaccination is amplified [29], which naturally includes news of serious side effects after vaccination for patients with chronic diseases [30, 31]. A recent longitudinal study also showed that people's concerns about the side effects and ineffectiveness of vaccination increased significantly over time [28]. Another important reason for this counterintuitive discovery may be related to the environment in which the participants were located. Specifically, during the current sampling period, China's ‘zero COVID-19’ policy was fully implemented, which created an environment with very few COVID cases [32]. Given the low risk of infection, the benefits of vaccination in terms of required protection were limited. In addition, the severe quarantine measures implemented for the whole community did not distinguish between vaccinated and unvaccinated populations, further reducing the benefits of vaccination [32]. Therefore, vaccination would only bring about possible side effects without any benefits of COVID-19 protection. Consequently, the motivation of vulnerable groups to vaccinate was relatively low, which is different from other countries. In these countries, COVID-19 cases are more common, and vulnerable populations need vaccination to protect them. The findings of our study prove that the ‘zero COVID-19’ policy has had a crowding-out effect on vaccination motivation. Another potential reason for the counterintuitive result is related to the vaccine rollout process in China. Rather than first vaccinating the most vulnerable, 18–59-year-old adults were the first to be vaccinated [33], giving the impression that elderly people and those who are sick should not be vaccinated. These may be the reasons for the decreasing vaccination rate for participants with poor subjective health status and chronic diseases. This also means that our findings might not apply to other countries with less strict COVID policies and that targeted interventions are needed for people with a poor subjective health status in China to increase vaccination coverage in countries with strict policies.

Consistent with previous studies, the psychological status of respondents, such as coping tendency, depression, neurasthenia and obesity, significantly affects vaccination willingness [16, 34]. In this study, the scores for negative coping tendency, depression, neurasthenia and obesity among the vaccinated were significantly lower than those of nonvaccinated people, and the score of positive coping tendency was significantly higher among vaccinated people than among the nonvaccinated. From the perspective of cognitive behavior theory, individual cognition directly affects all types of decisions, and psychological status is the direct response of individual cognitive thinking. Therefore, individuals with high scores for negative coping tendency, depression, anxiety and hypochondriasis will hesitate or even refuse to be vaccinated, while those with high scores for a positive coping style are more likely to be vaccinated.

Subjective health status is an individual's subjective evaluation and expectation of his or her health status. It has become one of the common health measurement methods in the world [26]. The evaluation method adopted in our study had only one question, and the answer was a simple 5-level evaluation. This enabled us to reduce the information acquisition time to the greatest extent and intuitively obtain the health status of individual subjective feelings. Our research showed that good subjective health status was a protective factor against vaccination behavior, and ROC analysis showed that subjective health status was the best index to predict the effectiveness of vaccination behavior among all the factors, with significant differences between the vaccinated and nonvaccinated groups. Similar to suffering from chronic diseases, we believe that the negative impact of poor subjective health status on vaccination behavior is also caused by the reasons mentioned above, such as the widespread negative information about the vaccine among the population, the crowding-out effect of the ‘zero-COVID’ policy [32] and the vaccine rollout process in China [33]. On the other hand, people with poor psychological status are more likely to hesitate when facing the choice of vaccination. Poor quality of life may also make people unable to find time to vaccinate. The index of subjective health status can reflect not only the status of physical diseases but also the psychological state and quality of life. Previous studies have also pointed out that subjective health status is a comprehensive index [35], which may contribute to its unique advantage in predicting vaccination behavior. In view of the obvious advantages of subjective health status in predicting vaccination behavior, it was further examined through subsequent logistic regression.

Logistic regression analysis showed that objective health status, that is, suffering from chronic diseases, was a risk factor for subjective health status, similar to previous studies [36]. Females and elderly people are also more likely to give pessimistic evaluations when evaluating their subjective health status than males and young people. The physical functions of elderly individuals are lower than those of young individuals to varying degrees. Due to social culture, women are more inclined to provide negative evaluations of their own health situation [37]. This is similar to men’s and women's evaluation of their appearance; generally, men have confidence in their appearance (even if their appearance is not outstanding in the eyes of others), while women still think their image is not perfect even if their image is good in the eyes of others [38]. Negative mental states such as depression, neurasthenia, obsession and hypochondriasis can also have a negative impact on subjective health. Based on the medical concept of integrating body and mind, psychological state is an important factor to evaluate individual health status. Moreover, people with a poor psychological state are more likely to practice negative thinking, which leads to the deterioration of their self-rated health status. Similarly, a positive coping style is beneficial to the estimation of individual health status. Through logistic regression, we found that the presence of chronic diseases, female sex, older age and negative mental states have a negative impact on subjective health status, which provides a specific intervention object for us to take intervention measures to improve the vaccination rate in the context of the current severe epidemic situation. Therefore, according to the above results, we need to pay special attention to patients with chronic diseases, elderly people, females and patients with mental diseases; fully mobilize the strength of government and community to monitor the vaccination of relevant populations; and intervene according to individual conditions. Our research focused on the final goal of current public health behaviors, vaccination behavior, and identified a simple and rapid prediction index that provides a simple and reliable marker for guiding vaccination work in the current context and informing interventions for people who refuse or hesitate to vaccinate.

It is worth mentioning that education is a protective factor for vaccination willingness or vaccination behavior at any time. Studies conducted before the outbreak of COVID-19 [26], studies on the COVID-19 outbreak [14], and studies of COVID-19 vaccination [8] have begun to show that education is beneficial to increasing the vaccination rate. Therefore, education can increase the vaccination rate at any time. Although the education level of all people cannot be significantly improved in the short term, in the long run, improving the education level of all people plays a vital role in the prevention and intervention of epidemic diseases.

The limitations of this study should be considered. First, the decision to vaccinate was provided by the respondents themselves, and the reliability of the data cannot be verified. Currently, the vaccination information of all citizens in China can be easily obtained through WeChat. In future research, respondents could be required to attach screenshots of vaccination information to ensure the reliability of the data. In addition, our study was a cross-sectional study. Therefore, we cannot obtain information about the dynamic changes between subjective health status and vaccination behavior with the change in the vaccination form, and we cannot determine causal relationships. Furthermore, the questionnaire was designed to be simple and easy to answer, so we could not evaluate other sociodemographic factors, such as personality, sleep status and daily habits.

## Conclusions

In summary, we found that 58.5% of respondents had been vaccinated before 4 June 2021. Moreover, our study found a simple and effective marker, subjective health status, that can predict vaccination behavior, which is beneficial to guide future epidemic prevention work. Suffering from chronic diseases, female sex, elderly age and negative emotional state were significant risk factors for subjective health status, which provides direction for the next specific intervention measures. In the future, longitudinal research should continue to pay attention to the relationship between subjective health status and vaccination behavior because people receive different information in different periods; different epidemic prevention policies and vaccine rollout process may have an effect on their judgment of the pros and cons of their own health status and whether they need vaccination.

## Supplementary Information

**Additional file 1:** **Table S1.** The scale of Psychological Questionnaire on Emergent Public Health Events (PQEPHE). **Table S2.** The scale of Simple Coping Style Questionnaire (SCSQ).

## Data Availability

The datasets used and analyzed in this study are available from the corresponding author on reasonable request.
