# Supplementary material for: Subjective health status: an easily available, independent, robust and significant predictive factor at the prometaphase of vaccination programs for the vaccination behavior of Chinese adults
Source: BMC Psychiatry. 2022 Mar 14;22:180. doi: 10.1186/s12888-022-03830-5 (PMC8920520; doi:10.1186/s12888-022-03830-5)
Supplement: Supplementary file 1 — Additional file 1: Table S1. The scale of Psychological Questionnaire on Emergent Public Health Events (PQEPHE). Table S2. The scale of Simple Coping Style Questionnaire (SCSQ). [file 12888_2022_3830_MOESM1_ESM.docx]

**Subjective health status: an easily available, independent, robust and significant predictive factor at the prometaphase of vaccination programs for the vaccination behavior of Chinese adults**

**Table S1.** The scale of Psychological Questionnaire on Emergent Public Health Events (PQEPHE).

**Table S2.** The scale of Simple Coping Style Questionnaire (SCSQ).

**Table S1.** The scale of Psychological Questionnaire on Emergent Public Health Events (PQEPHE).

| Items | No | Mild | Moderate | severe |
| --- | --- | --- | --- | --- |
| 1.Worry about being infected with yourself and your family | 0 | 1 | 2 | 3 |
| 2.Wash hands and scrub things repeatedly, but always feel that it is not clean enough | 0 | 1 | 2 | 3 |
| 3.Feel no spirit, dull brain, inattention and poor memory | 0 | 1 | 2 | 3 |
| 4.Feel your heart beating faster, sweating and blushing | 0 | 1 | 2 | 3 |
| 5.Less energy than before | 0 | 1 | 2 | 3 |
| 6.Mental fatigue is easy and difficult to recover | 0 | 1 | 2 | 3 |
| 7.No appetite, significant weight loss | 0 | 1 | 2 | 3 |
| 8.The brain is not as flexible as before | 0 | 1 | 2 | 3 |
| 9.When you encounter something related to the epidemic, you feel afraid and your heart beats faster | 0 | 1 | 2 | 3 |
| 10.Dizziness, palpitation, abdominal distension, constipation or diarrhea | 0 | 1 | 2 | 3 |
| 11.Headache, aching muscles all over | 0 | 1 | 2 | 3 |
| 12.In places where people gather, especially near the hospital, I feel nervous and nervous | 0 | 1 | 2 | 3 |
| 13.Not interested in anything | 0 | 1 | 2 | 3 |
| 14.Very concerned about any physical discomfort | 0 | 1 | 2 | 3 |
| 15.When you have symptoms associated with New Coronavirus infection, you suspect that you are infected | 0 | 1 | 2 | 3 |
| 16.Out of control | 0 | 1 | 2 | 3 |
| 17.It's easy to lose your temper when you feel annoyed | 0 | 1 | 2 | 3 |
| 18.I feel useless | 0 | 1 | 2 | 3 |
| 19.Knowing it won't help, but I can't control thinking and washing hands again and again | 0 | 1 | 2 | 3 |
| 20.Go to the hospital to see if you have been infected | 0 | 1 | 2 | 3 |
| 21.Poor sleep (difficulty falling asleep, dreaminess, lack of sleep after waking up, sleep rhythm disorder) | 0 | 1 | 2 | 3 |
| 1. Uncontrollably nervous or frightened | 0 | 1 | 2 | 3 |
| 23.Think of a dead man | 0 | 1 | 2 | 3 |
| 24.When you think of something related to the epidemic, you have no mind to do anything else | 0 | 1 | 2 | 3 |

**Scoring method**

Grade 0-3 is used for the test, that is, select "0" to count "0", and select 1, 2 and 3 to count 1, 2 and 3 in turn. The test is divided into five dimensions: depression, neurasthenia, fear, obsessive-compulsive anxiety and hypochondriasis. The scores of each dimension need to be calculated separately. The scoring method of a dimension score is to add the scores of the items contained in the dimension, and then divide by the number of items in the dimension. The specific scoring method is as follows:

Depression dimension score = (sum of scores of items 3, 5, 6, 7, 8 and 11) / 6

Neurasthenia dimension score = (sum of scores of items 13, 16, 17, 18 and 21) / 5

Fear dimension score = (sum of scores of items 1, 2, 9, 12 and 14) / 5

Score of obsessive-compulsive anxiety dimension = (sum of scores of items 4, 10, 19, 22, 23 and 24) / 6

Hypochondriac dimension score = (sum of scores of items 15 and 20) / 2

**Test introduction**

This test is the psychological questionnaire of sudden public health events compiled by scholar Gao Yan. The questionnaire was compiled after the SARS epidemic in 2003. It covers the common psychological and behavioral reactions of the public after public health emergencies, such as depression, anxiety, compulsion, fear and hypochondriasis. It can be widely used to evaluate the psychological status of people after public health emergencies. For the sake of understanding, this article revised the "sudden public health event" in the questionnaire to "New Coronavirus" or "epidemic situation".

**Table S2.** The scale of Simple Coping Style Questionnaire (SCSQ).

| Items | No | Mild | Moderate | severe |
| --- | --- | --- | --- | --- |
| 1. Relief through work, study or some other activities | 0 | 1 | 2 | 3 |
| 2. Talk to people and talk about your inner troubles | 0 | 1 | 2 | 3 |
| 3. Try to see the good side of things | 0 | 1 | 2 | 3 |
| 4.Change your mind and rediscover what's important in life | 0 | 1 | 2 | 3 |
| 5.Don't take the problem too seriously | 0 | 1 | 2 | 3 |
| 6.Stick to your position and fight for what you want | 0 | 1 | 2 | 3 |
| 7.Find out several different ways to solve the problem | 0 | 1 | 2 | 3 |
| 8.Seek advice from relatives, friends or classmates | 0 | 1 | 2 | 3 |
| 9. Change some of the original practices or some of your own problems | 0 | 1 | 2 | 3 |
| 10.Learn from others' methods of dealing with similar difficult situations | 0 | 1 | 2 | 3 |
| 11.Seek hobbies and actively participate in recreational and sports activities | 0 | 1 | 2 | 3 |
| 12.Try to restrain your disappointment, regret, back and anger | 0 | 1 | 2 | 3 |
| 13.Try to take a rest or vacation and put the problem (trouble) aside for the time being | 0 | 1 | 2 | 3 |
| 14.Relieve troubles by smoking, drinking, taking medicine and holding things | 0 | 1 | 2 | 3 |
| 15.Think that time will change the status quo. The only thing to do is to wait | 0 | 1 | 2 | 3 |
| 16.Try to forget the whole thing | 0 | 1 | 2 | 3 |
| 17.Rely on others to solve problems | 0 | 1 | 2 | 3 |
| 18.Accept the reality, because there is no other way | 0 | 1 | 2 | 3 |
| 19.Fantasize that some miracle may happen to change the status quo | 0 | 1 | 2 | 3 |
| 20. Comfort yourself | 0 | 1 | 2 | 3 |

**Application of SCSQ**

The questionnaire is a self-assessment scale, which adopts multi-level scoring. After each coping style item, there are four choices: do not use, occasionally use, sometimes use and often use (the corresponding scores are 0, 1, 2 and 3). The subjects choose one according to their own situation. The positive coping dimension consists of items 1-12, which mainly reflects the characteristics of positive coping, such as "seeing the good side of things as much as possible" and "finding out several different ways to solve problems"; The negative coping dimension consists of items 13-20, which mainly reflects the characteristics of negative coping, such as "relieving troubles through smoking and drinking" and "fantasy that some miracle may change the status quo". The results were the average scores of positive coping dimension and negative coping dimension. In clinical application, the scores of each answer should be further analyzed.
